# Investigating associations between intestinal alterations and parasite load according to *Bifidobacterium* spp. and *Lactobacillus* spp. abundance in the gut microbiota of hamsters infected by *Leishmania infantum*

**DOI:** 10.1590/0074-02760200377

**Published:** 2020-11-30

**Authors:** Fabine Correia Passos, Marcelo Biondaro Gois, Adenilma Duranes Sousa, Ananda Isis Lima de Marinho, Laura Corvo, Manoel Soto, Manoel Barral-Netto, Aldina Barral, Gyselle Chrystina Baccan

**Affiliations:** 1 Universidade Federal da Bahia, Instituto de Ciências da Saúde, Departamento de Bioquímica e Biofísica, Salvador, BA, Brasil; 2 Universidade Federal do Recôncavo da Bahia, Centro de Ciências da Saúde, Santo Antônio de Jesus, BA, Brasil; 3 Universidad Autónoma de Madrid, Facultad de Ciencias, Consejo Superior de Investigaciones Científicas, Centro de Biología Molecular Severo Ochoa, Departamento de Biología Molecular, Madrid, Spain; 4 Fundação Oswaldo Cruz-Fiocruz, Centro de Pesquisas Gonçalo Muniz, Salvador, BA, Brasil

**Keywords:** visceral leishmaniasis, L. infantum, intestinal tract, gut microbiota

## Abstract

**BACKGROUND:**

Visceral leishmaniasis (VL) is a tropical neglected disease with high associated rates of mortality. Several studies have highlighted the importance of the intestinal tract (IT) and gut microbiota (GM) in the host immunological defense. Data in the literature on parasite life cycle and host immune defense against VL are scarce regarding the effects of infection on the IT and GM.

**OBJECTIVES:**

This study aimed to investigate changes observed in the colon of *Leishmania infantum-infected* hamsters, including alterations in the enteric nervous system (ENS) and GM (specifically, levels of bifidobacteria and lactobacilli).

**METHODS:**

Male hamsters were inoculated with *L. infantum* and euthanised at four or eight months post-infection. Intestines were processed for histological analysis and GM analysis. Quantitative polymerase chain reaction (qPCR) was performed to quantify each group of bacteria: *Bifidobacterium* spp. (Bf) and *Lactobacillus* spp (LacB).

**FINDINGS:**

Infected hamsters showed histoarchitectural loss in the colon wall, with increased thickness in the submucosa and the mucosa layer, as well as greater numbers of intraepithelial lymphocytes. Forms suggestive of amastigotes were seen inside mononuclear cells. *L. infantum* infection induced changes in ENS, as evidenced by increases in the area of colonic enteric ganglia. Despite the absence of changes in the levels of Bf and LacB during the course of infection, the relative abundance of these bacteria was associated with parasite load and histological alterations.

**MAIN CONCLUSIONS:**

Our results indicate that *L. infantum* infection leads to important changes in the colon and suggest that bacteria in the GM play a protective role.

The intestinal tract (IT) and gut microbiota (GM) have been recognised as important to the maintenance of overall health. Dysbiosis, the pathological alteration of GM composition, is present in several diseases, and not only in disorders affecting the intestine, such as inflammatory bowel disease.^1^ ^,^ ^2^ ^,^ ^3^ ^,^ ^4^ Among the several bacteria species that compose the GM, the *Bifidobacterium* (Bf) and *Lactobacillus* (LacB) genera are considered indicators of good intestinal health, and exhibit important immunomodulatory properties that are related to their use as probiotics in the treatment of several diseases.^5^ ^,^ ^6^ In addition, the beneficial properties of Bf and LacB during parasitic infections have been demonstrated in experimental models of trypanosomiasis, toxoplasmosis and malaria.^7^ ^,^ ^8^ ^,^ ^9^

Studies have revealed that the GM plays important local and systemic functions, including the regulation of intestinal permeability, nutrient absorption and immune response. Intestinal homeostasis can be impacted by interactions occurring between GM, local leukocytes, enterocytes and the enteric nervous system (ENS).^10^ ^,^ ^11^

Visceral leishmaniasis (VL) is a neglected disease that affects over 20,000 people annually worldwide, mainly in underdeveloped countries.^12^ It is caused by parasites of the genus *Leishmania*, which are transmitted via the bite of vectors, insects of the *Phebotominae* family. Parasites multiply in several organs, including the spleen and liver. As the immune response mounted by the host is generally not effective in destroying all parasites, VL can be fatal without pharmacological treatment.

Although the parasite cycle and pathophysiological mechanisms of VL have been well-characterised, the roles played by the IT and GM in the development of VL have not been fully elucidated. Studies involving dogs naturally infected by *Leishmania* spp. have shown histopathological alterations in the jejunum and colon, as well as changes in the expression of immune receptors, cytokines and immune cells.^13^ ^,^ ^14^ ^,^ ^15^ ^,^ ^16^ Among all VL experimental models, the Golden Syrian hamster *Mesocricetus auratus* is considered a superior animal model to evaluate the parasite-host relationship, immunopathogenesis, drug discovery and vaccine development studies;^17^ ^,^ ^18^ ^,^ ^19^ ^,^ ^20^ ^,^ ^21^ ^)^ however these animals have not been used to investigate the effects of VL on IT and GM.

The present study aimed to investigate histopathological alterations in the IT of hamsters as well as changes in the levels of bifidobacteria (Bf) and lactobacilli (LacB) in the GM during the course of *L. infantum* infection.

## MATERIALS AND METHODS

*Animals, parasites and infection* - Male Syrian golden hamsters (*Mesocricetus auratus*), aged six to eight weeks, were obtained from the Animal Care Facility of the Gonçalo Moniz Institute/Oswaldo Cruz Foundation (IGM-FIOCRUZ). The local Institutional Review Board for Animal Experimentation approved all procedures involving animals (Health Sciences Institute of the Federal University of Bahia) (CEUA-ICS/UFBA 034/2012). All experiments involving animals were conducted in accordance with the guidelines established by the Colégio Brasileiro de Experimentação Animal (COBEA) and the Conselho Nacional de Controle de Experimentação Animal (CONCEA).

*L. infantum* (MCAN/BR/00/BA262) promastigotes were cultivated in Schneider’s insect Medium (Sigma Chemical Co., St Louis, MO, USA) supplemented with 20% inactivated fetal bovine serum, L-glutamine (2 mM), penicillin (100 U/mL) and streptomycin (100 μg/mL) at 23ºC for 5-7 days until parasites reached stationary phase. Parasites were then washed three times with saline at 3,000 rpm for 10 min, resuspended in saline and adjusted to 5 × 10^6^ per mL. Hamsters (n = 20) were inoculated with 1 x 10^5^ stationary-phase promastigotes intradermally in the ear. An identical number of uninfected hamsters were used as controls.

After infection, weekly evaluations included the evolution of body weight, the presence of clinical signs consistent with VL, including changes in hair appearance, paw arch and cachexia, as well as behavioral changes.

At four or eight months after infection, hamsters were euthanised by decapitation. Through vertical laparotomy, the liver, spleen and colon were removed, washed, measured and fixed. Stool samples were collected directly from the distal portion of the colon and stored at -80ºC until use.

*Parasite load determination* - Parasite load was evaluated by quantitative limiting dilution assay, as previously described by Lima et al.^22^ Briefly, the liver and spleen were aseptically removed from each hamster. Tissues were homogenised and diluted in Schneider’s insect cell culture medium (Sigma, St. Louis, MO) supplemented with 10% heat-inactivated fetal bovine serum, 100 U/mL of penicillin and 100 μg/mL of streptomycin. Homogenated samples were serially diluted on a 96-well microtiter plate and incubated for seven days at 23ºC. Dilutions corresponding to wells exhibiting positive growth were used to estimate parasite load in the samples.

*Histological processing and analysis* - A 1-cm ring sectioned from the colon of each hamster was fixed in buffered paraformaldehyde (10%) for 24 h. Segments were dehydrated in ascending series of ethyl alcohol, diaphanised in xylol and embedded in paraffin to obtain semi-serial cross-sections measuring 5 μm, which were then stained with Hematoxylin and Eosin.

Morphometric analysis was performed via images captured by a digital camera (Olympus® SC30, 3.0 Megapixel) coupled to an optical microscope (Olympus® BX43F - Minato-Ku, Japan). Measurements were obtained using Image-Pro Plus software version 4.5.0.29 (Media Cybernetics, Silver Spring, MD, USA). A 10× objective lens was used to capture images used to measure the total thickness of the intestinal wall, muscular tunic, submucosa, mucosa and the width and depth of crypts. Sixty-four measurements for each parameter were obtained throughout the circumference of each hamster’s colon.^23^ ^,^ ^24^ A 100× objective lens was used to capture images used to measure the height and width (at three points) of 80 enterocytes, as well as the smallest and largest diameters of nuclei in these cells.^23^ ^,^ ^24^ Images of the myenteric plexus and submucosal plexus ganglia were captured using a 40× objective to measure the area of 10 ganglia from each plexus in all hamsters. These results are expressed as the average area, in μm^2^, of the ganglion profiles of both the myenteric and submucosal plexuses.^25^

Intraepithelial lymphocytes (IELs) were quantified using an optical microscope with the aid of a 40× objective. The number of cells was obtained by manual counts among a total of 2,500 epithelial cells in each hamster, which permitted the assessment of the number of IELs/100 epithelial cells.^23^ ^,^ ^24^ ^,^ ^25^ ^,^ ^26^

Histopathological analysis was performed to determine the presence of forms suggestive of amastigotes,^15^ as well as histoarchitectural changes in the colon wall, by direct microscopy using objective lenses of 4×, 10×, 20×, 40× or 100×. In addition, inflammatory cell infiltrate was evaluated according to the following classification criteria: (i) Intensity: corresponding to the number of inflammatory cells observed - Absent (0-9 cells), Discreet (10-25 cells), Moderate (26-50 cells) or Intense (> 50 cells); (ii) Distribution: - Focal: a single infiltrate in the visual field; Multifocal, more than one infiltrate in the visual field; Diffuse: inflammatory cells diffusely distributed in a visual field.^14^ ^,^ ^15^ ^,^ ^23^ ^,^ ^24^ ^,^ ^27^ ^,^ ^28^

*Gut microbiota analysis* - DNA was extracted from stool samples using the PowerFecal® DNA Isolation Kit (MO BIO Laboratories, Carlsbad, CA, USA) in accordance with the manufacturer’s instructions. DNA concentrations were determined by absorbance at 260 nm, and purity was estimated by determining the A260 to A280 ratio. Measurements were performed on a microvolume NanoDrop^®^ ND-1000 spectrophotometer (NanoDrop Technologies, Wilmington, DE, USA). Purified DNA was stored at -20ºC until use.

The relative quantification of Bf and LacB was accomplished by quantitative polymerase chain reaction (qPCR) using group-specific 16S rRNA gene primers^29^ (Isogen Life Sciences, Netherlands) (Table I). A short segment of the 16S rRNA gene (200 bp) was specifically amplified by qPCR, using a conserved 16S rRNA-specific primer pair (Table I) to determine the total amount of commensal bacteria in the feces (the so-called ‘Eubacteria/Panbacteria’ group). Using the same genomic DNA from each sample, qPCR was completed using group-specific primers for the quantification of bacteria from *Bifidobacterium* spp. or *Lactobacillus* spp. (Table I).

**TABLE I:** Primers used to quantify bacterial populations by quantitative polymerase chain reaction (qPCR)

Bacterial group	Oligonucleotide sequence	Reference
Total bacteria	F: 5 ´ ACTCCTACGGGAGGCAGCAG 3 ´ R: 5 ´ ATTACCGCGGCTGCTGG 3 ´	^(29)^
*Bifidobacterium* spp*.*	F: 5´ TCGCGTCYGGTGTGAAAG 3´ R: 5´ RCCACATCCAGCRTCCAC 3´	^(29)^
*Lactobacillus* spp*.*	F: 5 ´ AGCAGTAGGGAATCTTCCA 3 ´ R: 5 ´ CACCGCTACACATGGAG 3’	^(29)^

F: forward; R: reverse.

Reactions were performed in glass capillary tubes using a LightCycler^®^ 2.0 thermocycler (Roche Applied Science, Alemanha). Reaction mixtures (10 μL) were composed of PowerUp™ SYBR™ Green Master Mix (2X) (Thermo‐Fisher Scientific, USA), 0.5 μL of each of the specific primers (forward and reverse) at a concentration of 10 μM/L and 2 μL of DNA template. A DNA concentration of 2 ng/µL was used in all reactions and the reaction volume was adjusted with MiliQ water to a final volume of 20 μL per capillary tube according to the manufacturer’s instructions. All reactions were performed in duplicate. Amplifications were performed using the following temperature profiles: one cycle at 95ºC (2 min), 40 cycles of denaturation at 95ºC (15 s), a primer annealing step 60ºC (1 min) and one final cycle at 95ºC (15 s). Fluorescent products were detected in the last step of each cycle. Melt curve analysis was performed following amplification to distinguish between targeted and non-targeted PCR products. Melting curves were obtained by slow heating from 65 to 95ºC, with fluorescence measurements taken after every 1ºC increase in temperature. A standard curve was generated for each set of primers. The qPCR amplification efficiency for all primer pairs was determined using the slope of linear regressions in a dilution series based on the following equation E = 10(-1/slope). Relative quantification was calculated by the 2 -ΔΔ Ct method^30^ (using the conserved 16S rRNA-specific primers (Total bacteria) as a reference gene). The results are expressed in terms of fold change over control.

*Statistical analysis* - Results are expressed as medians and interquartile ranges. The Kruskal-Wallis test was employed to analyse differences among more than two groups. Spearman’s correlation coefficient was applied to investigate associations between the relative abundances of bacterial groups, parasite loads and histological parameters. All statistical tests were performed using GraphPad Prism software version 5.01 (GrahPad Software, Inc.). The significance level adopted was 5%.

## RESULTS

**Fig. 1:**
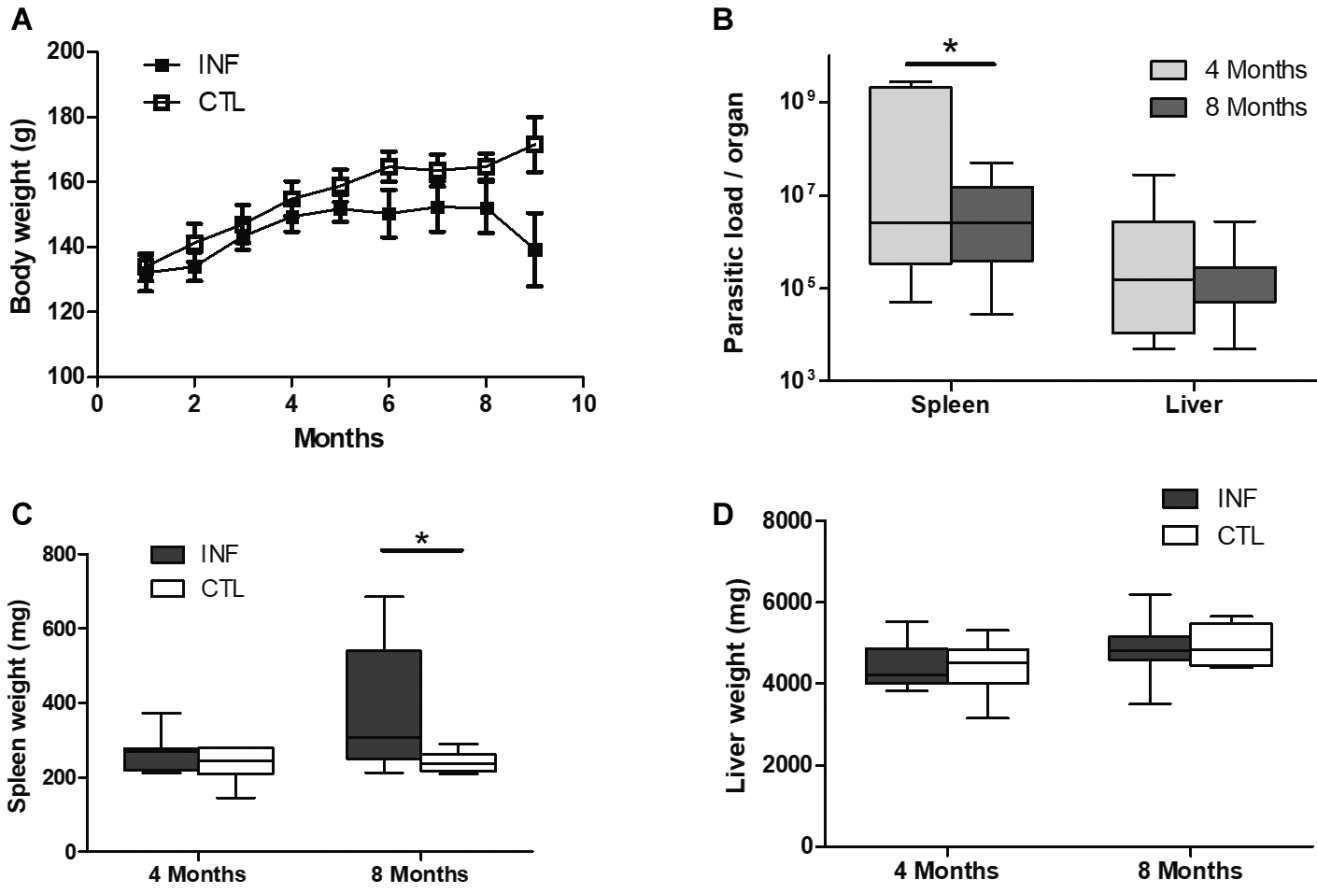
anatomic characteristics and parasite load in hamsters infected or not by *Leishmania infantum*. Body weight of infected (INF) and control (CTL) hamsters over eight months (A). Parasitic load was evaluated by limiting dilutions at four and eight months in the spleen and liver (B). Spleen and liver weight were measured at four and eight months post-infection (C-D). *p < 0.05.

*Detection of infection and parasite load* - Infected (INF) and uninfected hamsters (CTL) showed similar body weight evolution four months after infection, while differences were noted after eight months (Fig. 1A). Most animals did not present clinicopathological signs, and infection was confirmed by the parasite load in the spleen and liver (Figs 1B, C, D). Splenomegaly was detected only at eight months of infection (Fig. 1C). No differences were observed in liver size between the INF and CTL hamsters (Fig. 1D). Parasite load was determined at both four and eight month timepoints in the spleen and liver (Fig. 1B). As expected, splenic parasite load was found to be higher at four months than at eight months post-infection, yet this was not the case in the liver (Fig. 1B).

*Morphometric and histological alterations* - Morphometric changes were noted in the colon wall of infected hamsters at both four and eight months (Table II). Hypertrophy of the muscular layer was evidenced, with a significant increase of 14.32% after eight months of infection. Hypertrophy was also seen in submucosal thickness, with increases of 15.31% and 24.49% after four and eight months of infection, respectively. On the other hand, atrophy in the mucosa was observed, with a reduction of 16.65% after four months and 15.23% at eight months post-infection (Table II).

**TABLE II:** Morphometry of the colon of *Mesocricetus auratus* hamsters infected or not by *Leishmania infantum*

Parameter (µm)		Four months post-infection		Eight months post-infection	
		INF	CTL	INF	CTL
Muscular tunic	Thickness	280.1 (236.7 - 311.4)	268.4 (240.2 - 320.7)	346.3 (289.6 - 401.1)	322.2 (142.1 - 373.0)
Submucosa		98.1 (88.8 - 102.1)^*^	85.0 (75.0 - 96.4)	102.6 (90.4 - 115.1)^**^	80.9 (70.3 - 91.8)
Mucosa		338.9 (273.7 - 394.7)^*^	394.8 (309.8 - 454.4)	336.9 (276.7 - 378.3)	388.2 (339.4 - 474.5)
Crypts	Depth	159.2 (128.5-190.4)	216.4 (116.1 - 241.7)	98.1 (82.1 - 112.4)	103.2 (87.1 - 118.8)
	Width	54.5 (47.6-69.8)	75.6 (48.8 - 86.7)	47.9 (40.4 - 52.71)	43.3 (36.8 - 51.6)
Enterocytes	Height	18.7 (17.3 - 20.84)^**^	15.8 (13.7 - 18.8)	22.3 (18.6 - 24.1)	20.2 (17.6 - 22.4)
	Width	5.6 (4.7 - 6.4)	5.5 (4.6 - 6.4)	5.9 (5.4 - 6.7)^*^	5.5 (4.8 - 6.4)
Enterocyte nuclei	Largest-diameter	4.6 (3.7 - 5.4)	4.5 (3.6 - 5.4)	5.9 (5.1 - 6.6)^**^	4.6 (3.8 - 5.4)
	Smallest-diameter	3.3 (2.8 - 3.8)	3.1 (2.6 - 3.5)	3.4 (2.9 - 3.7)	3.5 (3.1 - 3.8)

Data expressed as medians (interquartile range). Results compared by the Kruskal-Wallis test (Results in bold indicate statistical differences compared to the control. CTL: control animals; INF: infected hamsters; *: p < 0.05; **: p < 0.001.

Enterocyte height increased by 15.08% after four months of infection and enterocyte width increased by 26.39% at the eight-month timepoint. In addition, alterations in core diameters were observed, with an 11.58% increase in the largest nucleus diameter seen at eight post-infection (Table II).

**Fig. 2:**
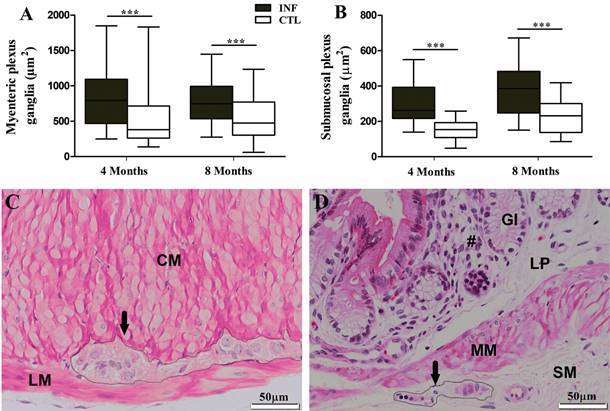
ganglion profiles (µm^2^) of the myenteric (A) and submucosal (B) plexuses. Histological staining of the myenteric plexus (black arrow) between the longitudinal (LM) and circular (CM) layers of the external musculature (C). Histological analysis of the submucosal plexus (black arrow) in the submucosa (D) of the colon wall in hamsters infected by *Leishmania infantum* four months post-infection, with a notable increase in inflammatory infiltrate (#) in the lamina propria (LP). MM: muscularis mucosa; SM: submucosa; GI: intestinal glands in the Crypts of Lieberkühn. Objective lens: 40x. Data expressed as medians (interquartile range). ***p *<* 0.001.

In addition to morphometric changes in the colon wall, *L. infantum* infection also caused neuroplastic changes in the myenteric and submucosal plexuses (Fig. 2). At four and eight months after infection, increases were observed in the area (µm^2^) of ganglions in both the myenteric and submucosal plexuses. In the myenteric plexus, an increase of 48.17% and 45.52% was noted at four and eight months, respectively (Fig. 2A). In the submucosal plexus, the area increased by 97.57% and 67.15% after four and eight months of infection, respectively (Fig. 2B). No significant changes were seen in the number of ganglia. In addition to neuroplastic changes, other important histopathological findings were the presence of diffuse inflammatory infiltrate in the lamina propria and within the ganglia of the submucosal plexus, characterising periganglionitis and ganglionitis, respectively (Fig. 2D).

**Fig. 3:**
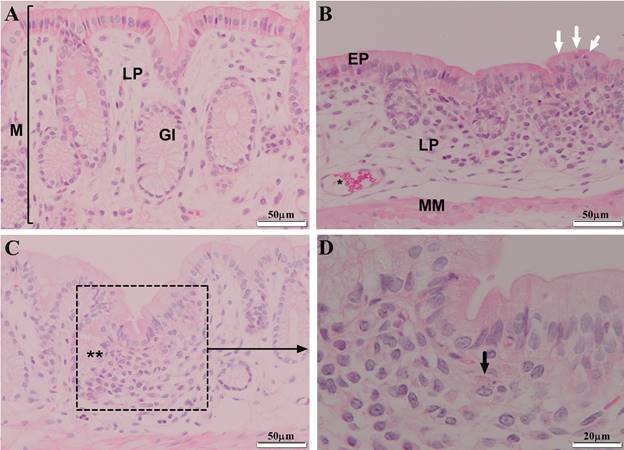
photomicrographs of cross-sections of the colon wall from uninfected (A) and infected hamsters (B-D) at eight months post-infection. (A) histoarchitecture of control animals; (B) infected hamsters demonstrating loss of wall histoarchitecture, presence of intraepithelial lymphocytes (white arrows), diffuse inflammatory infiltrate with a predominance of mononuclear cells in the lamina propria (LP), hypercellularity, presence of cells with plasma cell morphology and congested vessels (*); (C) hypercellularity and presence of focal inflammatory infiltrate (**); (D) inflammatory focus containing polymorphonuclear and mononuclear cells, as well as forms suggestive of amastigotes in the cytoplasm of a macrophage in the lamina propria (black arrow). Objective lens: 40x (A-C) and 100x (D). EP: epithelium; M: mucosa; LP: lamina propria; MM: muscularis mucosa; GI: intestinal glands in Crypts of Lieberkühn.

Histopathological analysis of the colonic mucosa revealed that *L. infantum* infection induced cellular and tissue changes at both the four and eight month timepoints (Fig. 3A-D). Forms suggestive of amastigotes were seen inside macrophages in the lamina propria (Fig. 3D). Semiquantitative histopathological analysis indicated a significant loss of colonic mucosa histoarchitecture, the presence of diffuse inflammatory infiltrate in the submucosa and lamina propria, moderate inflammatory infiltrate in the crypts, and a significant reduction in the number of goblet cells (Table III).

**TABLE III:** Semiquantitative histopathological analysis of colon mucosa in uninfected and infected hamsters

Parameter	Four months		Eight months	
	INF	CTL	INF	CTL
Histoarchitectural loss	2.0 (1.0 - 3.0)^*^	1.0 (1.0 - 1.75)	2.0 (2.0 - 3.0)^**^	1.0 (1.0 - 2.0)
Inflammatory infiltrate	2.0 (2.0 - 3.0)^**^	1.5 (1.0 - 2.0)	2.0 (2.0 - 3.0)^**^	1.5 (1.0 - 2.0)
Cryptitis	2.0 (1.0 - 2.0)^**^	1.0 (0.25 - 1.0)	2.0 (1.0 - 2.0)^**^	1.0 (1.0 - 2.0)
Goblet cells	1.0 (0.0 - 2.0)^**^	2.0 (2.0 - 3.0)	2.0 (0.0 - 3.0)	1.0 (1.0 - 3.0)

Scores expressed as medians (interquartile range). Results compared by the Kruskal-Wallis test (Results in bold indicate statistical differences compared to the control. CTL: control animals; INF: infected hamsters; *: p < 0.01; **: p < 0.001.

**Fig. 4:**
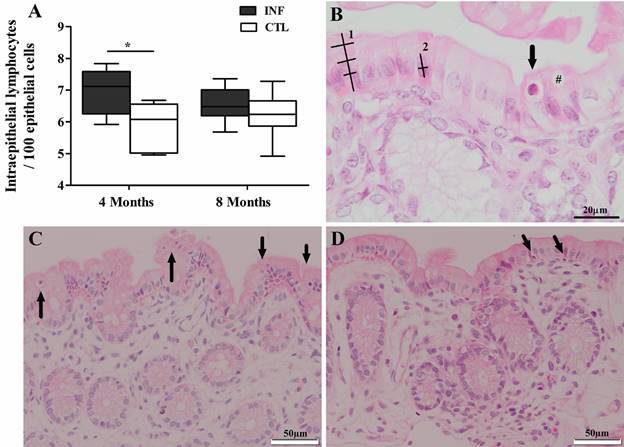
cellular alterations seen in the colon epithelium of uninfected (CTL) and infected (INF) hamsters. (A) Intraepithelial lymphocytes and epithelial cells quantified by optical microscopy (40x). Distribution of intraepithelial lymphocytes (arrows) in intestinal epithelium of CTL (B) and INF hamsters at four (C) and eight (D) months post-infection. (B) schematic measurement of height and width of enterocytes (1) and nuclei (2). #: goblet cell. Data expressed as medians (interquartile range). *p < 0.05.

In addition, our results indicate that *L. infantum* infection resulted in changes in the distribution of IELs, as evidenced by an increase of 18.35% in the colon mucosa of infected hamsters at four months compared to uninfected animals (Fig. 4).

**Fig. 5:**
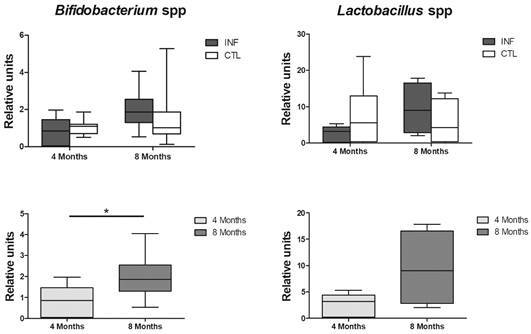
relative abundance of *Bifidobacterium* spp. and *Lactobacillus* spp. in the gut microbiota of hamsters uninfected (CTL) and infected by *Leishmania infantum* (INF). Lower panels represent bacterial abundance at four and eight months post-infection in INF animals only. Bacterial levels determined by quantitative polymerase chain reaction (qPCR). Data expressed as medians (interquartile range). *p < 0.05.

*Bifidobacteria and lactobacilli levels* - Relative levels of Bf and LacB were determined by qPCR (Fig. 5). The amplification efficiencies for the SYBR Green I assays were obtained by plotting the CT values against the target DNA starting quantity. Using the formula E = 10(-1/slope), the efficiencies for the individual assays ranged from 96.3-98.7 %.

No differences were found in the relative abundance of Bf or LacB when comparing between the INF and CTL hamsters, at either four or eight months of infection. However, in the INF group, a significant increase was observed in Bf levels at eight months compared to four months after infection (Fig. 5).

*Associations between parasite load, histological parameters and bifidobacteria and lactobacilli levels* - Bf levels were found to be negatively correlated with parasite load, as well as the area of myenteric and submucosal plexus ganglia (Table IV). The relative levels of LacB were also negatively associated with the area of the myenteric and submucosal plexus ganglia, and positively correlated with the loss of the histoarchitecture of the mucosa wall (Table IV).

**TABLE IV:** Correlations between disease parameters and relative abundance of *Bifidobacterium* spp and *Lactobacillus* spp

Parameter	Bifidobacteria		Lactobacilli	
	r	p	r	p
Body weight	0.3093	0.0799	-0.2545	0.2788
Spleen weight	-0.1409	0.4342	0.1662	0.4837
Liver weight	0.2276	0.2027	-0.1692	0.4757
Splenic parasite load	-0.5253	0.0252	-0.5030	0.1383
Liver parasite load	-0.2714	0.2760	-0.4182	0.2291
Loss of mucosal histoarchitecture	0.1610	0.1095	0.2047	0.0411
Cryptitis	0.2692	0.0067	0.1488	0.1577
Area of myenteric plexus ganglia	-0.2976	0.0001	-0.3177	< 0.0001
Area of submucosal plexus ganglia	-0.3693	< 0.0001	-0.3474	< 0.0001

r: Spearman’s correlation coefficient; p: statistical significance level.

## DISCUSSION

The present results demonstrate morphological alterations in the colon of hamsters infected by *L. infantum*, including histoarchitectural loss, increased inflammatory infiltrate and changes in ENS components. Despite similarities in the relative abundance of Bf and LacB between infected and control animals, these bacteria were nonetheless associated with lower parasite load and histological alterations in the colon of infected hamsters. Our results also indicate that *L. infantum* infection induced neuroplastic changes in ENS, as evidenced by increases in the area of ganglia in both the myenteric and submucosal plexuses.

Histological analysis revealed that *L*. *infantum* infection induced hypertrophy of the mucosal and atrophy of the submucosal layers of the Golden Syrian hamster colon. Animals infected by intradermal inoculation or by the bite of insect vectors have been used to study the immunopathological mechanisms of this disease, as well as in vaccine development, mainly due to the reproduction of clinicopathological aspects of human VL.^18^ ^,^ ^20^ ^,^ ^21^ ^,^ ^31^ Despite wide use of this experimental model, little has been reported regarding the morphometric changes in the IT induced by *L*. *infantum.* A study revealed that BALB/c mice inoculated intravenously with amastigotes of *L*. *infantum (chagasi)* exhibited atrophy of the ileum wall after 45 days of infection.^32^ In addition, hamsters and mice inoculated with other species of *Leishmania* also revealed morphometric changes in the IT.^23^ ^,^ ^24^

Alterations in the ENS have been described in other infections. Infection by *Toxoplasma gondii* was shown to result in a reduction in the number and area of ganglia in the myenteric plexus.^25^ Notably, *T. gondii* infection also induced enteric neuroplasticity.^33^ ^,^ ^34^ In contrast, neuroplastic changes caused by *Trypanosoma cruzi*, including those observed in the megaesophagus^35^ and megacolon,^36^ ^,^ ^37^ may compromise IT function. Infection by *T. cruzi* has been reported to result in increased colon size and diameter,^35^ as well as the death and hypertrophy of myenteric neurons,^38^ increased inflammatory infiltrate, myositis, periganglionitis, ganglionitis and elevated levels of pro-inflammatory cytokines.^35^

Regarding localised immune response, infection by *L. infantum* provoked an increase in immune cell migration to the lamina propria, which likely led to the observed loss of mucosal histoarchitecture and diffuse inflammation. This inflammatory infiltrate could be related to the cryptitis, periganglionitis and ganglionitis observed in this study. Moreover, the presence of suggestive forms of *L. infantum* amastigotes inside macrophages in intestinal mucosa corroborates these histopathological findings. Unfortunately, we were unable to identify the presence of amastigotes in the intestine of the infected hamsters evaluated herein; future studies should make an attempt to confirm this association. Previous studies in other experimental models of VL have shown the presence of *L. infantum* amastigotes in the liver, spleen and small and large intestine.^8^ ^,^ ^9^ ^,^ ^10^ ^,^ ^11^ ^,^ ^12^ ^,^ ^13^ ^,^ ^14^ ^,^ ^15^ ^,^ ^16^ ^,^ ^28^ ^,^ ^32^ ^,^ ^39^ Periganglionitis and ganglionitis, characterised by the infiltration of immune cells into the neural microenvironment, may occur primarily or secondarily to a wide variety of diseases.^36^ ^,^ ^37^ ^,^ ^40^ ^,^ ^41^ ^,^ ^42^ ^,^ ^43^ ^,^ ^44^ Inflammatory infiltrates in myenteric plexus ganglia compatible with periganglionitis and ganglionitis have been reported during infection induced by *L. infantum* in dogs^15^ and *L. (V.) braziliensis* in mice.^23^ These results corroborate our findings, since the presence of *Leishmania* sp. amastigotes correlates with tissue injury that could damage the ENS. Ganglionitis can cause inflammatory neuropathy and lead to the neuronal degeneration.^40^ ^,^ ^45^ Among the intestinal alterations reported in leishmaniasis are the formation of leishmaniotic granuloma, ulcerative colitis, degeneration in the crypt epithelium,^15^ ^,^ ^39^ chronic lymphadenitis and enteritis.^28^ A common element in all of these changes is the presence of diffuse inflammatory infiltrate in the intestinal wall containing mononuclear and polymorphonuclear cells.^14^ ^,^ ^15^ These findings lead us to suggest that the morphometric and neuroplastic alterations observed herein were likely triggered by the histopathological changes.

The integrity of the colonic epithelium was evaluated by measuring enterocyte height and width, as well as cellular nuclei, in addition to the distribution of intraepithelial lymphocytes. The number (18.3% increase) and distribution of IELs was observed to change in the colon of infected hamsters at four months. All studies reporting on tissue changes in IT arising from *Leishmania* sp infection detected changes in the ratio of lymphocytes to epithelial cells.^16^ ^,^ ^23^ ^,^ ^24^ In the megaesophagus and megacolon, neuronal degeneration has been associated with enteric ganglion invasion by cytotoxic T cells.^37^ Herein, the alterations seen in the myenteric and submucosal plexuses occurred simultaneously with histopathological changes, i.e. increases in the number of IELs and reduced goblet cells.

Injury to the submucosal plexus results in an imbalance between the immune system and intestinal epithelium,^46^ ^,^ ^47^ which is reflected in the GM.^10^ ^,^ ^11^ To investigate whether the GM could interact with the immune system and the ENS to reduce the impact associated with *L. infantum* infection, the relative abundance of *Bifidobacterium* spp. and *Lactobacillus* spp. was evaluated. These groups of bacteria were selected as they are considered indicators of good intestinal health that present important immunomodulatory properties and have had a long history of use as probiotics in the treatment of several diseases.^5^ ^,^ ^6^ In addition, several studies have evidenced the beneficial properties of Bf and LacB during parasitic infections in experimental models of trypanosomiasis, toxoplasmosis and malaria.^7^ ^,^ ^8^ ^,^ ^9^ Importantly, no changes were found in the relative abundance of Bf or LacB, perhaps due to low numbers of parasites in the hamster infection protocol, which did not lead to the development of severe disease. A recent meta-taxonomic analysis of GM in stool samples performed in Indian VL patients^48^ found no changes in GM compared to healthy individuals. Changes in GM and/or gut permeability could lead to microbial translocation and contribute to the rise in LPS plasma levels observed in VL patients, which are related to T-cell activation and increase in pro-inflammatory cytokines.^49^ Lamour et al.^50^ evaluated GM using next-generation sequencing in mice infected with *L. major* in two different experimental models of Cutaneous Leishmaniasis (CL) and detected marked changes in faecal bacterial composition after infections, which differed between the self-healing and non-healing mice.^50^ Another study investigated the role of GM in the development of CL using germ-free animals, reporting that the absence of GM at the beginning of infection may influence lesion size as well as immune response.^51^ ^,^ ^52^

Although our results did not indicate changes in the relative abundance of Bf and LacB, we did find associations between these bacteria present in the GM and parasite load as well as histological parameters, supporting the notion that these bacteria could have a host-protective role in infection. Furthermore, the observed decrease in spleen parasite load at eight months compared to the earlier 4-month timepoint post-infection, together with the inverse effect observed in relative Bf levels, points to the role of GM in parasitic control. While it is plausible that the relationship between relative Bf abundance and parasite load could be related to competitive exclusion and immunomodulatory aspects,^5^ ^,^ ^53^ we were unable to determine a causal relationship, since a higher parasite load could induce decreases in Bf levels as well.

The present study is limited by the fact that changes in the relative abundance of other groups of bacteria were not evaluated, nor in the diversity of the composition of the GM.

*In conclusion* - Our results suggest that *L. infantum* infection results in morphometric, neuroplastic and histopathological changes in the colon in Golden Syrian hamsters. While experimental infection was not found to promote changes in the relative abundance of Bf and LacB, these bacteria could be associated with the immunological control of parasite load and colon injury, which warrants further study. Our findings are consistent with those in the literature indicating the importance of the IT and GM in the host defense response.
